# Evaluation of Corrosion Residual Life Prediction Methods for Metal Pipelines

**DOI:** 10.3390/ma15165624

**Published:** 2022-08-16

**Authors:** Lili Zuo, Chunlei Zeng, Xingqiao Hu, Shengjie Du, Yun Zhao, Fan Fei

**Affiliations:** 1 National Engineering Laboratory for Pipeline Safety, MOE Key Laboratory of Petroleum Engineering, Beijing Key Laboratory of Urban Oil and Gas Distribution Technology, China University of Petroleum (Beijing), Beijing 102249, China; 2 China Waterborne Transport Research Institute, Beijing 100088, China; 3 Beijing Gas Group Co., Ltd., Beijing 100035, China; 4 National Petroleum and Natural Gas Pipe Network Group Beijing Pipeline Co., Ltd., Beijing 100101, China

**Keywords:** corroded pipeline, residual life prediction, anti-corrosion, BP neural network, inspection data

## Abstract

The analysis of the basic characteristics of various research methods is highly needed to predict the residual life of the pipeline accurately, help managers understand the operational risks, and provide a reference for developing pipeline transportation and maintenance inspection plans and anti-corrosion measures. Based on a comprehensive investigation of the existing research on the residual life of the pipeline, this paper finds that the current mainstream life prediction method, based on historical statistical data, has the shortcomings of inconsistent modeling methods, inconsistent basic data, and a lack of comparative evaluation among methods. Moreover, considering the in-depth study of BP neural network modeling, grey theory modeling, time series modeling, and exponential smoothing modeling, optimal prediction models using different methods based on the same historical data are established. These optimal modeling methods are discussed, and the feasible modeling path for the accurate prediction of the pipeline’s residual life is given by comparing the prediction accuracy of each model. In addition, the findings serve as a guide for developing an anti-corrosion strategy by highlighting the contribution of the prediction results of the residual life to pipeline decision-making. By comparison, it is found that the accuracy of the four prediction models is as follows: the grey theory prediction model, the exponential smoothing prediction model, the BP neural network prediction model, and the time series prediction model, from high to low, respectively.

## 1. Introduction

A great number of statistics show that corrosion defects are the main factors causing pipeline accidents. Corrosion severely restricts pipeline capacity and increases the expenditure of capital. For high-pressure, burnable, and detonatable oil and gas pipelines, once corrosion failure occurs, the consequences are very serious. Therefore, it is of great significance to master the corrosion condition and residual life of pipelines to facilitate transportation, maintenance plans and anticorrosive measures.

In recent years, there has been an increasing emphasis on the prediction of the residual life of corroded pipelines. Current research methods mainly fall into three categories. In category 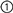, the pipeline corrosion rate is regarded as an uncertain and random value, and the residual life of the corroded pipeline has been predicted by studying the distribution model of the maximum corrosion depth data, which mainly includes GEV distribution, Gumbel distribution, Frechet distribution, and Weibull distribution. Zhang X S et al. [1] established a residual life prediction model of corroded oil and gas pipelines based on improved GEV distribution. Firstly, the Markov Chain Monte Carlo (MCMC) method was used to estimate the parameters of the GEV distribution function and determine the type of extreme value distribution. When the graphic test was found to be reasonable, then the distribution could be used to predict the maximum corrosion depth. Secondly, a corrosion allowance prediction model was established based on the reliability and safety of the pipeline. Finally, according to the data of the maximum corrosion depth, corrosion allowance and annual service life of the pipeline, the relationship index model of the three was established to predict the residual life of the pipeline. Based on Gumbel distribution, Zhang X S et al. [2] processed the maximum corrosion depth data randomly selected from the inspection data of an oil and gas pipeline, established the prediction model of the maximum corrosion depth of the pipeline, and then estimated the value of the parameters of the prediction model with the Markov Chain Monte Carlo (MCMC) method and predicted the possible maximum corrosion depth through the model. Hence, based on the obtained corrosion depth, critical corrosion depth and pipeline service life and other data, the relationship index model between the three was established to predict the residual life of oil and gas pipelines. Wang R et al. [3,4] realized the scientific evaluation and prediction of the corrosion status and operation of offshore oil and gas pipelines by utilizing Frechet extreme value distribution to establish the prediction model of the maximum corrosion depth of offshore oil and gas pipelines, combined it with the Monte Carlo (MC) method to estimate the parameter value of the prediction model and predict the possible maximum corrosion depth, and analyzed and predicted the maximum probability of pipe wall corrosion through the Markov Chain model. Similarly, F. Caleyo et al. [5] used the Monte Carlo simulations to study the probability distributions of external corrosion pit depth and pit growth rate in underground pipelines and combined a predictive pit growth model with the observed distributions of the model variables in a range of soils. Depending on the pipeline age, any of the three maximal extreme value distributions, i.e., Weibull, Fréchet or Gumbel, can arise as the best fit to the pitting depth and rate data.

Category 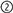 is based on the reliability theory, using the ultimate limit state function to establish the mathematical probability model of pipeline failure, and predict the residual life of the pipeline and the cumulative failure probability at a given time. Shuai J [6] regarded various factors affecting the residual life of pipelines as random variables with different distributions, and established a mathematical probability model to predict pipeline failure. Using this model, the effects of corrosion rate, defect depth, pipe wall thickness, and working pressure on the reliability of the pipeline were studied. The corrosion rate obtained from the analysis can reasonably predict the safety status of the whole pipeline. Yu S R et al. [7] established a probability model for predicting the residual life of pipeline corrosion based on the shell-92 deterministic model. The Monte Carlo method was used to calculate the residual life of the pipeline and its cumulative distribution function, and the parameter sensitivity analysis was carried out. The main parameters affecting the corrosion residual life of the buried pipeline and their variation with the service time were discussed. Alma Valor et al. [8] derived different corrosion rate distributions from various corrosion growth models and used these to perform reliability analyses of underground pipelines. Hu Q F et al. [9] proposed a nonlinear prediction model for the maximum corrosion depth of gas pipelines under different sample independence by using the Bayesian estimation method based on the probability distribution of model parameters, and solved the model by using the MCMC method. Luo J H et al. [10] used the size data of nearly 1000 corrosion overhaul defects of a pipeline over the years to calculate the corrosion rate distribution of the pipeline and establish a probability distribution model of corrosion rate. Based on the reliability theory, the corrosion residual life of the pipeline was predicted by using the limit defect size data determined by the corrosion residual strength evaluation. Additionally, three deterministic and probabilistic models were introduced by Markus R. Dann et al. [11] to account for the sizing bias present in in-line inspection data for corrosion growth analysis. 

Category 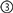 is based on historical statistical data such as corrosion rate and remaining thickness, using different modeling theories and methods to explore the change rules in the historical data, and extrapolating the data in the timeline to predict the residual life of the pipeline. The Modeling methods and theories used in this kind of research mainly include the BP neural network modeling method, gray theory modeling method, exponential smoothing method and time series prediction method. Using the grey theory, Yu X C et al. [12] proposed effectively predicting the corrosion rate via the complex mapping relationship between the corrosion rate and the corrosion influencing factors in the water injection pipeline. At the same time, to improve the prediction accuracy, the standard GM (1,1) model was reasonably improved, in order to predict the change trend of the corrosion rate with time. Wang H T et al. [13] used the cubic exponential smoothing method to establish the prediction model of the pipeline corrosion rate, fitted and predicted the corrosion rate data, and obtained the most reasonable weight coefficient in the prediction model α. Then, through the comparative analysis with the primary exponential smoothing method and the quadratic exponential smoothing method, it was concluded that the cubic exponential smoothing method has higher prediction accuracy and that the predicted value is consistent with the actual value. Kevin S et al. [14] utilized historical excavation and recoat information to identify static defects and quantify systemic bias between inspections. To reduce differences in reporting and the analyst interpretation of the recorded magnetic signals, novel analysis techniques were employed to normalize the data sets against each other. The resulting uncertainty of the corrosion growth rates was then further reduced by deriving and applying a regression model to reduce the effect of the different sizing models and the identified systemic bias. Liu X N [15] established the quantitative relationship between corrosion residual life and corrosion rate, coefficient of variation, corrosion allowance and reliability, and obtained the calculation formula to determine the corrosion residual life. Zhang X S et al. [16] analyzed the feasibility of building a grey theory model, established a GM (1,1) model with optimized parameters, changed the initial conditions of the model, and predicted the corrosion depth of submarine pipelines. According to the predicted corrosion depth, the Markov model was used to quantitatively analyze the future corrosion state of the submarine pipeline and predict its residual life. Xiao W et al. [17] determined the corrosion risk prediction method suitable for the Tahe Oilfield by comparing the application scope, reliability and economy of five common pipeline corrosion risk prediction methods. The classical BP neural network algorithm was optimized with the help of a genetic algorithm, which effectively improves the accuracy and reliability of the BP neural network. Yao Q [18] used the historical data collected on site, combined with the characteristics of the corrosion problem, and adopted the time series model to predict the corrosion rate. On this basis, he used the Monte Carlo method to evaluate the residual life of the equipment, and judged the residual service life of the equipment through the statistics of the corrosion failure probability of the equipment, so as to obtain the residual life of the equipment.

Compared with category 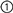, category 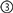 can reflect the relationship of corrosion degree with time more directly. Meanwhile, compared with category 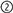, category 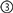 can directly provide the residual life of the pipeline under the current corrosion condition, but not the reliability or failure probability of the residual life. At present, most of the researches who prefer category 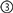 of methods focus on the selection of modeling methods and theories. However, the lack of comparative studies using the same basic statistical data and different modeling methods and theories makes it challenging to objectively and quantitatively assess the benefits and drawbacks of the prediction accuracy of each method. Based on the same basic statistical data, this study uses a variety of modeling methods and theories to establish residual life prediction models. By comparing the accuracy of the predictions, it then discusses the applicability and reliability of each modeling method and theory.

## 2. Prediction Method of Residual Life of Metal Pipe 

**Figure 1:**
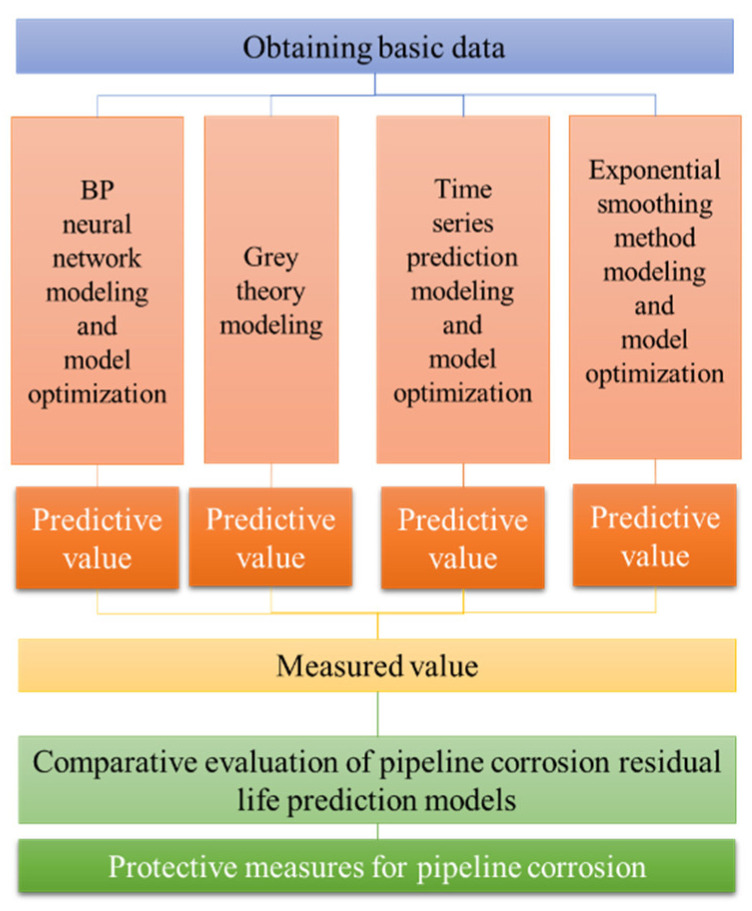
Technical route of residual life prediction method for metal pipelines.

This study evaluates the application effects of the BP neural network method, grey theory method, exponential smoothing method and time series prediction methods in metal pipeline corrosion. The research ideas can be described as follows: establishing the prediction model by using each modeling method, then optimizing the parameters of each model based on the same basic statistical data, and finally getting the optimal prediction model under each modeling method. The optimal prediction model is used for prediction, and the prediction results are compared with the measured values to evaluate the applicability and reliability of each method. Then, the application strategy of corrosion life prediction results in corrosion prevention is discussed (Figure 1).

For a given metal pipe, its corrosion depth in the *i*-th period can be expressed by the average corrosion rate:

(1) $$V_{i}=\frac{\delta_{i}}{T_{i}}=\frac{d_{i,1}-d_{i,2}}{T_{i}}$$

where $V_{i}$ is the average corrosion rate in the *i*-th period, mm/a; $\delta_{i}$ is the corrosion depth in the *i*-th period, mm; *T_i_* is the length of the *i*-th period, a; $d_{i,1}$ is the pipe wall thickness at the beginning of the *i*-th period, mm; and $d_{i,2}$ is the pipe wall thickness at the end of the *i*-th period, mm.

After corrosion for a long time (*N* time cycles), the remaining wall thickness (*d*) of the pipeline is:

(2) $$d=d_{0}-\overset{N}{\underset{i=1}{\sum }}\delta_{i}=d_{0}-\overset{N}{\underset{i=1}{\sum }}(V_{i}T_{i})$$

where *d*_0_ is the initial wall thickness of the pipeline, mm.

As can be seen from Equation (2), the following two routes can be used to predict the residual life of corroded pipelines under the condition that the minimum allowable thickness of pipelines is determined (which can be calculated by the ultimate bearing capacity and other methods [10]).

Method of predicting residual wall thickness: By obtaining the remaining pipe wall thickness at fixed periodic points, such as the routine inspection of the pipe wall’s thickness once a year, modeling methods and theories are further used to establish a prediction model to predict the change value of the wall thickness in subsequent cycles, and the calculation results are compared with the minimum allowable thickness of the pipe to determine the residual life of the corroded pipe [15].

Method of predicting average corrosion rate: According to Equations (1) and (2), it can be seen that there is a definite relationship between the corrosion depth and the average corrosion rate in a certain period. Using a method similar to the prediction of residual wall thickness, the average corrosion rate within a fixed period is taken as the prediction object, and the corrosion depth and residual corrosion thickness are obtained through transformation. The residual life of the corroded pipeline is obtained after comparing it with the minimum allowable thickness of the pipeline [12,17,18].

The above two methods are similar. The method of predicting the average corrosion rate is selected in this study—that is, to predict the change rule of the average corrosion rate in each period of the corrosion pipeline in the subsequent operation. Therefore, the main factors determining the prediction accuracy are the applicability and accuracy of modeling methods and theories.

## 3. Modeling Methods and Theories

### 3.1. The BP Neural Network Modeling

**Figure 2:**
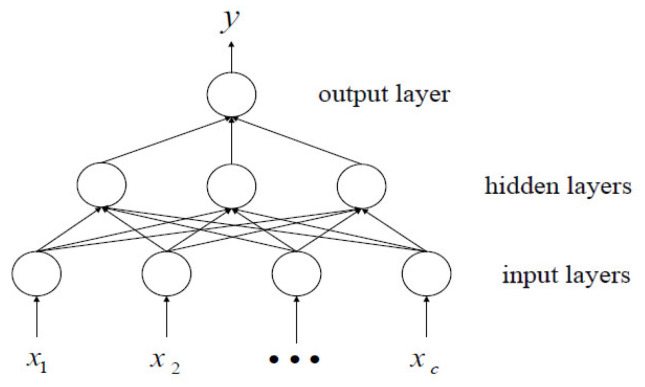
Structure diagram of multilayer perceptron neural network.

Among the many neural networks, the multilayer perceptron neural network is one of the most popular ones. Such networks typically consist of one input layer, one or more hidden layers, and one output layer (Figure 2). Each layer contains multiple neurons, and the input layer receives input signals *x*_1_, *x*_2_, …, *x_c_*, while the output layer returns the output result *y*.

**Figure 3:**
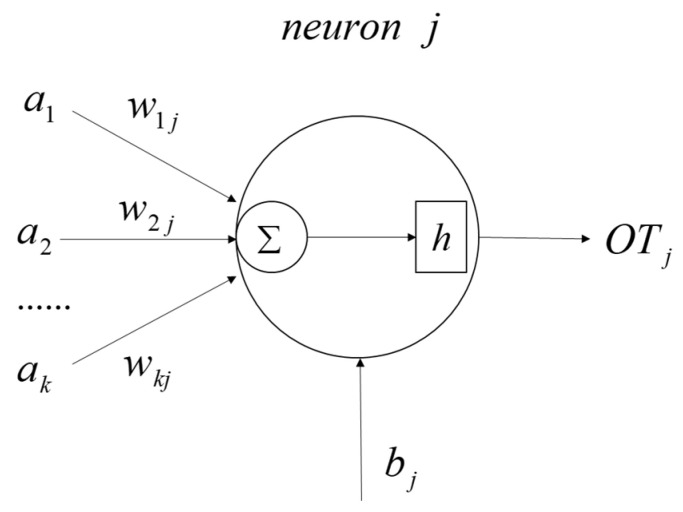
Input and output schematic diagram of neuron *j*.

In the multi-layer perceptron neural network, each neuron is a signal transmission node and can receive multiple input signals $a_{1}$,$a_{2}$,…,$a_{n}$. For neuron *j*, the weight of signal *a_k_* is *w_kj_*. The weighted results are summed and the threshold $b_{j}$ is added as the total input of the neuron (Figure 3). Finally, the transfer function *h* is applied to the total input to obtain the neuron’s output *OT_j_*:

(3) $$OT_{j}=h\left(\underset{k}{\sum }w_{kj}a_{k}+b_{j}\right)$$

where *h* is the transfer function, which is used to establish the relationship between the neuron input and output.

The transfer function can introduce nonlinear factors into neurons, so the neural network can approximate any nonlinear function. Thus, this neural network can be applied to nonlinear models. In practical applications, there are many optional transfer functions, and the sigmoid function is more commonly used:

(4) $$h(S_{j})=\frac{1}{1+e^{S_{j}}}$$

where $S_{j}=\underset{k}{\sum }w_{kj}a_{k}+b_{j}$ is the total input of neuron *j*.

When the ownership value and threshold value of each neuron in the neural network are determined, the functional relationship between the total input of the neural network $x_{1}$,$x_{2}$,…,$x_{c}$ and the final output y is uniquely determined, which is the mapping relationship between input and output determined by the neural network:

(5) $$y=l\left(x_{1},x_{2},\ldots ,x_{c}\right)$$

where l is the mapping relationship between total input $x_{1},x_{2}$,…$,x_{c}$ and output *y*.

The implementation steps of the BP neural network algorithm are as follows:

Step 1: set the number of nodes, transfer function, weight and threshold of each neuron in the input layer, hidden layer and output layer.

Step 2: input training samples and calculate the results of the hidden layer units and output layer units.

Step 3: calculate the network output error, and then back propagate to the input layer by layer through the hidden layer, and allocate the error to all units of each layer.

Step 4: adjust the weight and threshold of each unit of each layer according to the error signal back propagated.

Step 5: check whether the total error of the network meets the accuracy requirements. If so, the training ends; If not, return to step 2.

### 3.2. The Grey Theory Modeling

Grey theory refers to the fuzziness, randomness and uncertainty of a system. The establishment model of grey theory system is called grey theory model, which is called the GM model for short. GM model can reveal the characteristics and the laws of continuous development and change hidden in the system. The grey theory prediction model generally refers to the GM (1,1) model. $x^{\left(0\right)}=\left[x^{\left(0\right)}\left(1\right),x^{\left(0\right)}\left(2\right),\ldots ,x^{\left(0\right)}\left(n\right)\right]$; $x^{\left(1\right)}$ is the 1-AGO sequence of $x^{\left(0\right)}$.

(6) $$x^{\left(1\right)}=\left[x^{\left(1\right)}\left(1\right),x^{\left(1\right)}\left(2\right),\ldots ,x^{\left(1\right)}\left(n\right)\right]$$

The relationship between $x^{\left(0\right)}$ and $x^{\left(1\right)}$ is:

(7) $$x^{\left(1\right)}\left(p\right)=\sum_{m=1}^{p}x^{0}\left(m\right)$$

GM (1,1) model is defined as:

(8) $$x^{\left(0\right)}\left(p\right)+uz^{\left(1\right)}\left(p\right)=f$$

where $z^{\left(1\right)}\left(p\right)$ is the background value of GM (1,1) model.

The whitening differential equation of GM (1,1) model is:

(9) $$\frac{dx^{\left(1\right)}\left(t\right)}{dt}+ux^{\left(1\right)}\left(t\right)=f$$

The integration of Equation (9) on the interval [*p*−1, *p*] can be obtained as follows:

(10) $$\overset{p}{\underset{p-1}{\int }}\frac{dx^{\left(1\right)}\left(t\right)}{dt}dt+u\overset{p}{\underset{p-1}{\int }}x^{\left(1\right)}\left(t\right)dt=f$$

(11) $$x^{\left(1\right)}\left(p\right)-x^{\left(1\right)}\left(p-1\right)+u\overset{p}{\underset{p-1}{\int }}x^{\left(1\right)}\left(t\right)dt=f$$

(12) $$x^{\left(0\right)}\left(p\right)+u\overset{p}{\underset{p-1}{\int }}x^{\left(1\right)}\left(t\right)dt=f$$

According to the second mean value theorem of integration, if f is monotonic on [a, b], ∃ ξ, st $\overset{b}{\underset{a}{\int }}f\left(x\right)g\left(x\right)dx=f\left(a\right)\overset{\xi }{\underset{a}{\int }}g\left(x\right)dx+f\left(b\right)\overset{b}{\underset{\xi }{\int }}g\left(x\right)dx$

(13) $$\begin{array}{cc} ∴\overset{p}{\underset{p-1}{\int }}x^{\left(1\right)}\left(t\right)dt & =x^{\left(1\right)}\left(p-1\right)\left(\xi -\left(p-1\right)\right)+x^{\left(1\right)}\left(p\right)\left(p-\xi \right)\\ & =\alpha x^{\left(1\right)}\left(p-1\right)+\left(1-\alpha \right)x^{\left(1\right)}\left(p\right) \end{array}$$

In Equation (13), α=ξ−(p−1)

Take α=0.5, then $z^{\left(1\right)}\left(p\right)=\overset{p}{\underset{p-1}{\int }}x^{\left(1\right)}\left(t\right)dt=\frac{1}{2}x^{\left(1\right)}\left(p-1\right)+\frac{1}{2}x^{\left(1\right)}\left(p\right)$

Furthermore, Equation (12) can be reduced to $x^{\left(0\right)}\left(p\right)+uz^{\left(1\right)}\left(p\right)=f$, that is, $-uz^{\left(1\right)}\left(p\right)+f=x^{\left(0\right)}\left(p\right)$

It can be obtained by the least square method that:

(14) $$u=\frac{\sum_{p=2}^{n}z^{\left(1\right)}\left(p\right)\sum_{p=2}^{n}x^{\left(0\right)}\left(p\right)-\left(n-1\right)\sum_{p=2}^{n}z^{\left(1\right)}\left(p\right)x^{\left(0\right)}\left(p\right)}{\left(n-1\right)\sum_{p=2}^{n}z^{\left(1\right)}{\left(p\right)}^{2}-{\left(\sum_{p=2}^{n}z^{\left(1\right)}\left(p\right)\right)}^{2}}$$

(15) $$f=\frac{\sum_{p=2}^{n}x^{\left(0\right)}\left(p\right)\sum_{p=2}^{n}z^{\left(1\right)}{\left(p\right)}^{2}-\sum_{p=2}^{n}z^{\left(1\right)}\left(p\right)\sum_{k=2}^{n}z^{\left(1\right)}\left(p\right)x^{\left(0\right)}\left(p\right)}{\left(n-1\right)\sum_{p=2}^{n}z^{\left(1\right)}{\left(p\right)}^{2}-{\left(\sum_{p=2}^{n}z^{\left(1\right)}\left(p\right)\right)}^{2}}$$

From Equation (9), the form of the solution of the albino differential equation is:

(16) $$x^{\left(1\right)}\left(t\right)=Ce^{-u\left(t-1\right)}+\frac{f}{u}$$

The initial value condition is: $x^{\left(1\right)}\left(0\right)=x^{\left(0\right)}\left(1\right)=x^{\left(1\right)}\left(1\right)$. By substituting the initial value condition into Equation (16), we obtain:

(17) $$x^{\left(1\right)}\left(t\right)=\left(x^{\left(0\right)}\left(1\right)-\frac{f}{u}\right)e^{-u\left(t-1\right)}+\frac{f}{u}$$

The discrete solution of the differential equation is:

(18) $$x^{\left(1\right)}\left(p+1\right)=\left(x^{\left(0\right)}\left(1\right)-\frac{f}{u}\right)e^{-up}+\frac{f}{u}$$

The ${\hat{x}}^{\left(1\right)}$ sequence can be obtained by substituting calculations *u* and *f* into Equation (18), and the reduced value (predicted value) ${\hat{x}}^{\left(0\right)}$ sequence can be obtained by further using Equation (7).

### 3.3. The Time Series Prediction Modeling

A time series refers to a group of observed or recorded data arranged in chronological order, commonly represented as *X*_1_, *X*_2_,…, *X_n_*. A sequence contains all information about the historical behavior of the system that produced the sequence. The basic idea of the time series prediction method is to establish a mathematical model which can accurately reflect the dynamic dependence relationship contained in the time series and predict the future behavior of the system based on the finite length of operation records (observation data). The moving average method is a common method among time series methods, mainly including the primary moving average method and secondary moving average method.

The moving average method refers to the average of a fixed number of data each time, in chronological order step by step. For each period, the data of the previous period should be discarded and the data of a new period should be added, and then the average should be carried out. In other words, use (*x_t_* + *x_t_*_−1_ + … + *x_t_*_−*N*+1_)/*N* to predict *x_t_*_+1_. To obtain the best prediction accuracy, the MSE of past data prediction is often used as the criterion to select the number of terms N in the first moving average method:

(19) $$\mathrm{MSE}=\frac{1}{K-N}\sum_{t=N+1}^{K}{\left(Y_{t}-{\hat{Y}}_{t}\right)}^{2}$$

The second moving average is a moving average of the actual value based on a moving average. The quadratic moving average can establish the linear trend prediction model:

(20) $$X_{t+T}=A_{t}+B_{t}T$$

(21) $$A_{t}=2M_{t}^{\left(1\right)}-M_{t}^{\left(2\right)}$$

(22) $$B_{t}=\frac{2\left(M_{t}^{\left(1\right)}-M_{t}^{\left(2\right)}\right)}{N-1}$$

where *T* is the predicted time point in the future; $X_{t}$ is the predicted value at time point t; $M_{t}^{\left(1\right)}$ is the primary moving average at time t, $M_{t}^{\left(1\right)}=\frac{1}{N}\overset{N-1}{\underset{j=0}{\sum }}X_{t-j}$; and $M_{t}^{\left(2\right)}$ is the quadratic moving average at time t, $M_{t}^{\left(2\right)}=\frac{1}{N}\overset{N-1}{\underset{j=0}{\sum }}M_{t-j}^{(1)}$. By solving Equations (21) and (22) and substituting them into Equation (20), the predicted value can be obtained.

### 3.4. The Exponential Smoothing Method Modeling

The exponential smoothing method is a suitable method for simple time series analysis and short- and medium-term forecasts. According to the different smoothing times, it can be divided into primary exponential smoothing, secondary exponential smoothing, cubic exponential smoothing and high-order exponential smoothing. High-order exponential smoothing is rarely used. According to [13], compared with the primary and secondary exponential smoothing methods, the cubic exponential smoothing method has a higher accuracy in pipeline corrosion rate prediction. The cubic exponential smoothing method is mainly used in this study.

Cubic exponential smoothing is an exponential smoothing method based on quadratic exponential smoothing. Regarding the calculation of the primary exponential smoothing value and the quadratic exponential smoothing value, the cubic exponential smoothing value is calculated by the following formula:

(23) $$V_{d}^{\left(3\right)}=\alpha V_{d}^{\left(2\right)}+\left(1-\alpha \right)V_{d-1}^{\left(3\right)}$$

If the time series has a conic trend change and the future is predicted to change according to this trend, the conic trend prediction model can be established:

(24) $$X_{d+D}=\beta_{d}+\gamma_{d}D+\delta_{d}D^{2}$$

(25) $$\beta_{d}=3V_{d}^{\left(1\right)}-3V_{d}^{\left(2\right)}+V_{d}^{\left(3\right)}$$

(26) $$\gamma_{d}=\frac{\alpha }{2{\left(1-\alpha \right)}^{2}}\left[\left(6-5\alpha \right)V_{d}^{\left(1\right)}-2\left(5-4\alpha \right)V_{d}^{\left(2\right)}+\left(4-3\alpha \right)V_{d}^{\left(3\right)}\right]$$

(27) $$\delta_{d}=\frac{\alpha^{2}}{2{\left(1-\alpha \right)}^{2}}\left[V_{d}^{\left(1\right)}-2V_{d}^{\left(2\right)}+V_{d}^{\left(3\right)}\right]$$

where $V_{d}^{\left(1\right)}$,$V_{d}^{\left(2\right)}$ and $V_{d}^{\left(3\right)}$ are the first-, second- and third-order smoothing index values, respectively; α is the smoothing coefficient, 0 < α < 1; *D* is the predicted time point in the future; and $X_{d}$ is the predicted value at time point *d*.

By calculating Equations (25)–(27), $\beta_{d}$, $\gamma_{d}$ and $\delta_{d}$ are calculated and substituted into Equation (24) to obtain the predicted value.

## 4. Case Analysis

### 4.1. Data Sources

**Figure 4:**
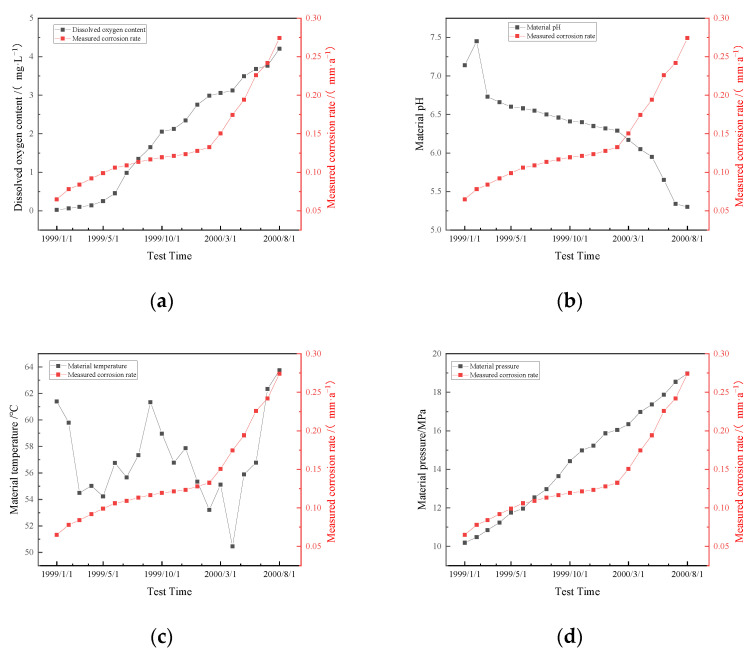

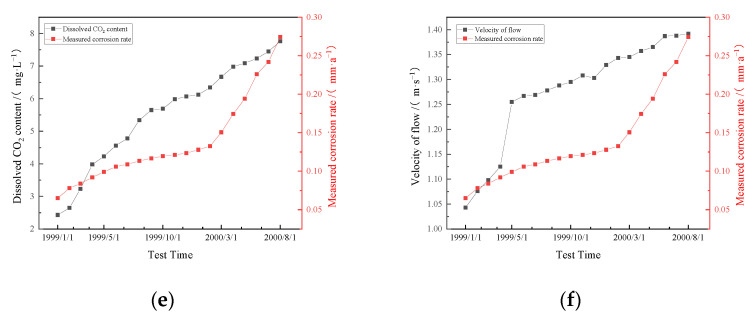
(**a**) Dissolved oxygen content; (**b**) Material pH; (**c**) Material temperature; (**d**) Material pressure; (**e**) Dissolved CO_2_ content; (**f**) Velocity of flow Relationship between various data and corrosion rate in oil field.

In this study, the corrosion life prediction is indirectly given by predicting corrosion rate. To compare the prediction accuracy of different modeling methods, the measured corrosion data of metal pipelines in an oil field in [12] (Figure 4 and Table 1) are selected for discussion and research.

Table 1 shows a total of 20 groups of measured corrosion data of an oil field pipeline from 1 January 1999 to 1 August 2000. The types of data detected include the test time, dissolved oxygen content, pH value of transmission medium, operating temperature, operating pressure, dissolved CO_2_ content, the flow rate of the transmission medium and the measured corrosion rate. Based on the measured corrosion rate, this paper compares and analyzes the accuracy of four different corrosion life prediction models.

Figure 4 clearly shows that dissolved oxygen content, material pressure, dissolved CO_2_ content and velocity of flow are positively correlated with correlation rate, material pH is negatively correlated with correlation rate, and material temperature has no significant correlational trend with correlation rate.

### 4.2. The BP Neural Network Modeling Optimization and Prediction

A prediction model of corrosion rate is established by using a multi-layer sensory neural network. Six parameters including dissolved oxygen content, material pH value, material temperature, material pressure, dissolved CO_2_ content and flow rate are taken as the input variables of the model, and corrosion rate is taken as the output of the model. Therefore, the neural network has six nodes in the input layer and one node in the output layer.

The prediction accuracy of the multilayer neural network model is affected by the number of hidden layers, nodes of hidden layers, transfer function and training algorithm. To obtain the optimal neural network model with the highest prediction accuracy, a trial calculation method is adopted to determine the optimal choice. In the trial calculation, the first 15 groups of data in Table 1 are taken as the training set of the model, and the last 5 groups of data are taken as the verification set. The MSE of the predicted corrosion rate and the measured corrosion rate of the verification set are calculated to determine the optimal neural network prediction model. Two kinds of neural networks, either with one hidden layer and two hidden nodes or one hidden layer and four hidden nodes, are selected for trial calculation, and the Levenberg-Marquardt BP (L-MBP for short) and Bayers normalized BP algorithm are applied to each algorithm, respectively. The transfer function is double tangent S-type and S-type. 

MATLAB programming modeling is used to predict the validation set (Table 2 and Table 3). It can be seen that when the BP neural network uses one hidden layer and four hidden nodes, the training algorithm uses L-MBP and the transfer function is S-type, the prediction accuracy of the model is the highest. The mean square error (MSE) of the BP neural network predicted value and measured value is 0.000299.

### 4.3. The Grey Theory Modeling and Prediction

Based on the first 15 groups of measured data in Table 1, the measured time interval is one month, and the original corrosion rate data are calculated first:

$$x^{\left(0\right)}=\left[x^{\left(0\right)}\left(1\right),x^{\left(0\right)}\left(2\right),\ldots ,x^{\left(0\right)}\left(15\right)\right]$$

Then, the original data column are accumulated once to generate

$$x^{\left(1\right)}=\left[x^{\left(1\right)}\left(1\right),x^{\left(1\right)}\left(2\right),\ldots ,x^{\left(1\right)}\left(15\right)\right]$$

Further calculate the MEAN sequence:

$$z^{\left(1\right)}=\left[z^{\left(1\right)}\left(2\right),\;z^{\left(1\right)}\left(3\right),\ldots ,z^{\left(1\right)}\left(n\right)\right]$$

Substitute the above $x^{\left(0\right)}$, $x^{\left(1\right)}$ and $z^{\left(1\right)}$ into Equation (14) and Equation (15) to obtain *u* and *f*; Further substitute the obtained *u* and *f* into Equation (18), and the simulation calculation value can be obtained through calculation:

$$\begin{array}{cc} \left\{{\hat{x}}^{\left(1\right)}(k)\right\} & =\left[{\hat{x}}^{\left(1\right)}(1),{\hat{x}}^{\left(1\right)}(2),\ldots ,{\hat{x}}^{\left(1\right)}(20)\right]\\ & =(0.065,0.0773,0.0821,0.0908,0.0112,0.1177,0.1221,0.1335,0.1399,0.1421,0.1467,\\ & \;0.1489,0.1500,0.1594,0.1645,0.1833,0.1900,0.2149,0.2311,0.2557) \end{array}$$

The mean square error (MSE) between the predicted value and the measured value is 0.000135.

### 4.4. Modeling Optimization and Prediction of Time Series Prediction Method

The time series prediction method can adopt the first moving average method and the second moving average method, and the second moving average method is suitable for the multi-series continuous prediction in this example. In the calculation of the quadratic moving average method, it is necessary to calculate the prediction results when *N* = 3, *N* = 5 and *N* = 7, and select the optimal *N* value and prediction model according to the comparison of the mean square error between the prediction results and the measured values. The specific method is to calculate $M_{t}^{\left(1\right)}$ and $M_{t}^{\left(2\right)}$ according to the value of N, then use Equations (21) and (22) to calculate $A_{t}$ and $B_{t}$ (the first 15 groups of data are used for modeling, the last five groups of data are used for verification and comparison, and the value of T is 15), and finally substitute it into Equation (20) to calculate the predicted value (Table 4). It can be seen that when *N* = 3, the prediction accuracy of the established time series prediction model is the highest, and the mean square error between the predicted value and the measured value is 0.003744.

### 4.5. Exponential Smoothing Modeling Optimization and Prediction

According to [13], compared with the primary and secondary exponential smoothing methods, the cubic exponential smoothing method has a higher accuracy in pipeline corrosion rate prediction. Thus, only the cubic exponential smoothing method is studied. The main parameter affecting the prediction accuracy is the smoothing coefficient α. To obtain the optimal prediction model, α is set as 0.3, 0.5 and 0.7 for trial calculation, and the optimal value of α and the prediction model are selected according to the comparison between the prediction result of the model and the measured value of the mean square error. The specific method is to calculate $V_{d}^{\left(1\right)}$, $V_{d}^{\left(2\right)}$ and $V_{d}^{\left(3\right)}$ according to the value of α; Equations (25) to (27) are used to calculate $\beta_{t}$, $\delta_{t}$ and $\gamma_{t}$ (the first 15 groups of data are used for modeling, the last 5 groups of data are used for verification and comparison, and *t* is set to 15). Then, substitute it into Equation (24) to calculate the predicted value (Table 5). It can be seen that when α = 0.7, the cubic exponential smoothing model has the highest prediction accuracy, and the mean square error (MSE) between the predicted value and the measured value is 0.000241.

### 4.6. Comparison and Analysis of Prediction Models

**Figure 5:**
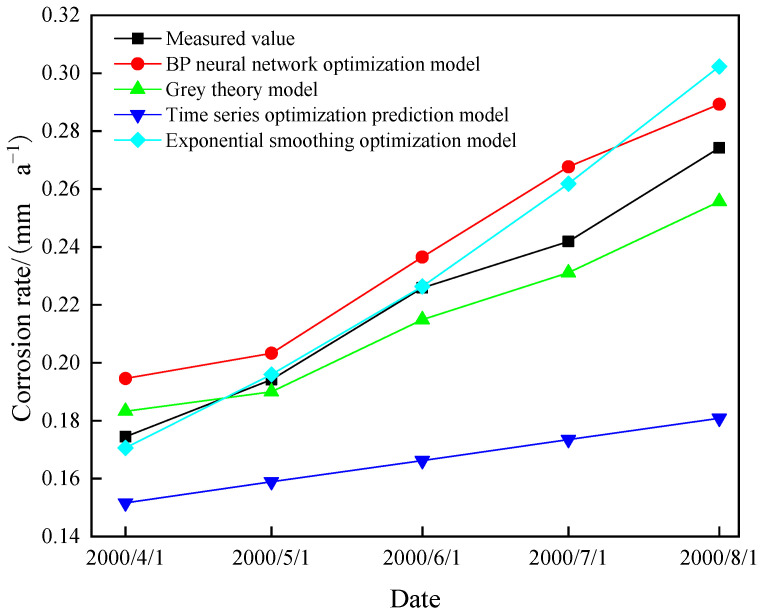
Comparison curves of predicted and measured values of each model.

From the above calculation, the optimal prediction models of each modeling method can be obtained. When the prediction results are compared to the measured values (Figure 5 and Table 6), it is clear that the prediction accuracy of the grey theory model is higher than that of other modeling methods, and this method is more accurate in predicting the corrosion rate of the corroded pipeline in the subsequent operation. According to the predicted corrosion rate, the annual corrosion depth and remaining wall thickness are calculated, which are compared with the minimum allowable thickness of the pipeline to determine the residual life of the pipeline.

### 4.7. Summary

The grey theory model was used to predict the corrosion rate of the corroded pipeline during the subsequent operation process. Hence, the annual corrosion depth and residual wall thickness were calculated, which were compared with the minimum allowable thickness of the pipeline to determine its residual life. When developing a corrosion repair strategy, the enterprise should not only focus on the pipeline’s residual life, but also on the required operation time, the cost and technical difficulty of anticorrosion repair, the repair effect and stability, and the use environment of the pipeline [19,20,21,22,23,24,25].

For corroded pipelines, enterprises need to consider the following repair strategies: decide whether to repair, determine the best time node for repairing, and adopt a repair plan. Common repair solutions include direct replacement, external card maintenance, HDPE composite structure pipeline repair technology, pipeline welding reinforcement technology [19], pipeline carbon fiber reinforcement technology, flip lining repair technology, etc. Regarding the above-mentioned prediction results of the residual life of the corroded pipeline, as well as the daily inspection and the publicity along the pipeline, one or more of the above-combined repair strategies can be considered to reduce the cost and process of pipeline repair while achieving smooth operation under the premise of satisfying safety measures.

## 5. Conclusions

Regarding the comprehensive investigation of the existing residual life of corroded pipelines, the advantages and disadvantages of modeling prediction methods based on historical statistical data are compared and evaluated.

(1) The existing modeling methods, each with their own benefits and drawbacks, can be used to predict the residual life of corroded pipelines. However, the neural network modeling method can intuitively reflect the relationship between corrosion rate and corrosion influencing factors, and the established model’s basic theory is more reasonable.
(2) The grey theory prediction model is suitable for short-, medium-, and long-term prediction and has the advantages of small samples, lack of sample regularity, low computational workload, and high accuracy. It can fully mine the internal information in a small amount of data and produce a more reasonable prediction from fewer data. Comparative analysis reveals that the grey theory method has good applicability and reliability.
(3) The goal of the time series prediction method is to establish a mathematical model in a way that, given the system’s finite number of operation records (observation data) accurately captures the time series’ dynamic dependencies. The prediction value always remains at its previous level, but it sometimes struggles to accurately predict the future trend. As a result, the accuracy is lower when compared to other prediction models.
(4) The exponential smoothing prediction model is easy to predict, and it only needs to select one model parameter α, and can automatically identify and adjust changes in data patterns. It has a better short-term prediction effect following the gray theory prediction method.

## Figures and Tables

**Table 1:** Measured values of pipeline corrosion data in an oil field.

Serial Number	Test Time	Dissolved Oxygen Content (mg·L^−1^)	Material pH	Material Temperature °C	Material Pressure MPa	Dissolved CO_2_ Content (mg·L^−1^)	Velocity of Flow (m·s^−1^)	Measured Corrosion Rate (mm·a^−1^)
1	1 January 1999	0.023	7.14	61.4	10.19	2.43	1.043	0.065
2	1 February 1999	0.065	7.45	59.8	10.48	2.65	1.076	0.078
3	1 March 1999	0.104	6.73	54.5	10.85	3.23	1.098	0.084
4	1 April 1999	0.143	6.66	55.03	11.24	3.98	1.125	0.092
5	1 May 1999	0.254	6.60	54.23	11.75	4.23	1.255	0.099
6	1 June 1999	0.456	6.58	56.76	11.96	4.56	1.267	0.106
7	1 July 1999	0.987	6.55	55.67	12.54	4.78	1.269	0.109
8	1 August 1999	1.345	6.50	57.34	12.97	5.34	1.278	0.1134
9	1 September 1999	1.654	6.46	61.34	13.65	5.65	1.288	0.1167
10	1 October 1999	2.054	6.41	58.97	14.43	5.69	1.295	0.1195
11	1 November 1999	2.124	6.40	56.78	14.98	5.98	1.308	0.1213
12	1 December 1999	2.345	6.35	57.87	15.23	6.07	1.303	0.1234
13	1 January 2000	2.753	6.32	55.34	15.87	6.12	1.329	0.1279
14	1 February 2000	2.987	6.29	53.22	16.05	6.34	1.343	0.1326
15	1 March 2000	3.057	6.17	55.12	16.34	6.67	1.345	0.1506
16	1 April 2000	3.125	6.05	50.45	16.98	6.98	1.357	0.1745
17	1 May 2000	3.492	5.95	55.9	17.37	7.09	1.365	0.1942
18	1 June 2000	3.682	5.65	56.78	17.87	7.23	1.387	0.2259
19	1 July 2000	3.769	5.34	62.34	18.54	7.45	1.388	0.2419
20	1 August 2000	4.208	5.30	63.76	18.98	7.76	1.392	0.2742

**Table 2:** Results of the neural network with 1 hidden layer and 2 hidden nodes.

Training Algorithm	Transfer Function	Trial Calculation Results (mm·a^−1^)					Mean Square Error between Predicted value and Measured Value
		1 April 2000	1 May 2000	1 June 2000	1 July 2000	1 August 2000	
L-M BP	Double tangent S-type	0.1845	0.2042	0.2133	0.2788	0.2932	0.000416
	S-type	0.1611	0.1783	0.2011	0.2222	0.2488	0.000416
Bayers normalized BP	Double tangent S-type	0.1533	0.1712	0.2922	0.2356	0.2867	0.001114
	S-type	0.1595	0.1732	0.2011	0.2207	0.2511	0.000453

**Table 3:** Experimental results of the neural network with 1 hidden layer and 4 hidden nodes.

Training Algorithm	Transfer Function	Trial Calculation Results (mm·a^−1^)					Mean Square Error between Predicted Value and Measured Value
		1 April 2000	1 May 2000	1 June 2000	1 July 2000	1 August 2000	
L-M BP	Double tangent S-type	0.1443	0.1574	0.1958	0.2114	0.2444	0.000998
	S-type	0.1946	0.2033	0.2365	0.2677	0.2893	0.000299
Bayers normalized BP	Double tangent S-type	0.1855	0.2133	0.2484	0.2709	0.3021	0.000522
	S-type	0.1456	0.1638	0.2099	0.2233	0.2658	0.000486

**Table 4:** Time series prediction modeling results.

*N*	Parameter				Predictive Value (mm·a^−1^)					MSE
	$M_{15}^{\left(1\right)}$	$M_{15}^{\left(2\right)}$	$A_{15}$	$B_{15}$	1 April 2000	1 May 2000	1 June 2000	1 July 2000	1 August 2000	
3	0.137033	0.129733	0.144333	0.007300	0.1516	0.1595	0.1662	0.1735	0.1808	0.003744
5	0.131160	0.122540	0.139780	0.004310	0.1441	0.1484	0.1527	0.1570	0.1613	0.005665
7	0.127429	0.115263	0.139594	0.004055	0.1436	0.1477	0.1518	0.1558	0.1599	0.005819

**Table 5:** Exponential smoothing prediction modeling results.

α	Parameter						Predictive Value (mm·a^−1^)					MSE
	$S_{t}^{\left(1\right)}$	$S_{t}^{\left(2\right)}$	$S_{t}^{\left(3\right)}$	$a_{15}$	$b_{15}$	$c_{15}$	1 April 2000	1 May 2000	1 June 2000	1 July 2000	1 August 2000	
0.3	0.131778	0.120023	0.110064	0.145328	0.006742	0.000165	0.1522	0.1595	0.1670	0.1749	0.1832	0.003588
0.5	0.139569	0.132255	0.126884	0.148825	0.01217	0.000971	0.1620	0.1771	0.1941	0.2130	0.2340	0.000783
0.7	0.144631	0.139926	0.136147	0.150261	0.017814	0.002519	0.1706	0.1960	0.2264	0.2618	0.3023	0.000241

**Table 6:** The predicted value and measured value of each model and their mean square error.

Model	Predictive Value (mm·a^−1^)					Absolute Error (mm·a^−1^)					MSE
	1 April 2000	1 May 2000	1 June 2000	1 July 2000	1 August 2000	1 April 2000	1 May 2000	1 June 2000	1 July 2000	1 August 2000	
BP neural network optimization model	0.1946	0.2033	0.2365	0.2677	0.2893	0.0201	0.0091	0.0106	0.0258	0.0151	0.000299
Grey theory model	0.1833	0.1900	0.2149	0.2311	0.2557	0.0088	0.0042	0.0110	0.0108	0.0185	0.000135
Time series optimization prediction model	0.1516	0.1589	0.1662	0.1735	0.1808	0.0229	0.0353	0.0597	0.0684	0.0934	0.003744
Exponential smoothing optimization model	0.1706	0.1960	0.2264	0.2618	0.3023	0.0039	0.0018	0.0005	0.0199	0.0281	0.000241
Measured value/(mm·a^−1^)	0.1745	0.1942	0.2259	0.2419	0.2742	/	/	/	/	/	/

## Data Availability

Restrictions apply to the availability of these data. Data was obtained from CNKI (China national knowledge internet) and are available [https://kns.cnki.net/kcms/detail/detail.aspx?dbcode=CJFD&dbname=CJFD2003&filename=FSYF200302001&uniplatform=NZKPT&v=Radetct_Cp3NgIrtYlV8aEY1ZYZPw0BjQw-Wi6z8TdXybk8PUgxXymeujCmfX10S, accessed on 18 May 2022] with the permission of CNKI.
